# Anti-hnRNP B1 (RA33) Autoantibodies Are Associated with the Clinical Phenotype in Russian Patients with Rheumatoid Arthritis and Systemic Sclerosis

**DOI:** 10.1155/2014/516593

**Published:** 2014-05-04

**Authors:** Aleksey Maslyanskiy, Natalya Lazareva, Polina Olinek, Peter Schierack, Christian Hentschel, Juliane Cuccato, Dimitrios P. Bogdanos, Sergey V. Lapin, Dirk Roggenbuck

**Affiliations:** ^1^Department of Rheumatology, Almazov Medical Research Centre, 197341 St. Petersburg, Russia; ^2^Laboratory of Autoimmune Diagnostics, St. Petersburg Pavlov State Medical University, 197022 St. Petersburg, Russia; ^3^Eichwald Department of Therapy and Rheumatology, Mechnikov Northwestern State University, 191015 St. Petersburg, Russia; ^4^Faculty of Science, Brandenburg University of Technology Cottbus-Senftenberg, Großenhainer Straße 57, 01968 Senftenberg, Germany; ^5^R/D, Medipan GmbH, Dahlewitz, 15827 Berlin, Germany; ^6^Institute of Liver Studies, Division of Transplantation Immunology and Mucosal Biology, King's College London School of Medicine at King's College Hospital, London SES 9RJ, UK

## Abstract

Heterogeneous nuclear ribonucleoproteins (hnRNPs) are potent autoantigenic targets in systemic autoimmune rheumatic diseases (SARD). Loss of tolerance to the RA33 complex consisting of hnRNP A2 and its alternatively spliced variants B1 and B2 has been the interest of rheumatologists. A novel ELISA for the detection of anti-hnRNP B1 autoantibodies has been developed to investigate the prevalence thereof in 397 patients with SARD, including patients with rheumatoid arthritis (RA), spondyloarthropathy (SPA), juvenile chronic arthritis, systemic lupus erythematosus (SLE), systemic sclerosis (SSc), and Sjögren's syndrome (SS), in comparison to 174 controls. Anti-hnRNP B1 autoantibodies were significantly more prevalent in patients with SARD than controls (47/397, 11.8% versus 2/174, 1.1%; *P* < 0.001). In particular, anti-hnRNP B1 were found more frequently in the disease cohorts than in the controls and were present in 24/165 (14.5%) patients with RA, 6/58 (10.3%) SPA, 11/65 (16.9%) SSc, and 4/50 (8.0%) SLE. In RA patients, anti-hnRNP B1 autoantibodies correlated significantly with C-reactive protein levels and erythrocyte sedimentation rate, while in patients with SSc it was associated with features of arterial wall stiffness and presence of hypertension. Anti-hnRNP B1 autoantibodies occur in SARD and seem to be correlated with distinct clinical characteristics in patients with RA and SSc.

## 1. Introduction

Heterogeneous nuclear ribonucleoproteins (hnRNPs) are nucleoplasmic molecules interacting with premessenger ribonucleic acid (pre-mRNA) and partake in the processing thereof [1]. In general, hnRNPs contain at least one RNA recognition motif representing the RNA-binding domain. Furthermore, they can play a role in various other important cellular mechanisms like DNA repair, telomere elongation, chromatin remodelling, and translocation, as well as nuclear-cytoplasmic shuttling, translation, and regulation of proteins. Loss of immunological tolerance to hnRNP has been reported in several systemic autoimmune rheumatic diseases (SARD) [2]. Hitherto, 30 major hnRNPs with the terminology A1 through U have been described. Of them particularly hnRNP A1, A2, B, C, H, I, and R could be demonstrated as autoantigenic targets in SARD [3].

Autoreactivity to the RA33 complex mainly consisting of autoantibodies to hnRNP A2 and its alternatively spliced variants B1 and B2 has been demonstrated in patients with rheumatoid arthritis (RA) as early as 1989 [4]. Thus, the respective autoantibody was referred to as anti-RA33 because of its reaction with a 33 kDa antigen by immunoblotting employing nuclear extracts from HeLa cells. Apart from immunoblotting, enzyme-linked immunosorbent assay (ELISA) has been employed mainly to test for anti-RA33, but experimental testing has led to inconsistent results amongst studies. Nevertheless, several reports revealed a prevalence of about 30% for anti-B1/A2 hnRNP autoantibodies in patients with RA [5]. However, those autoantibodies have been also found in patients with systemic lupus erythematosus (SLE) and other SARD [6, 7]. Such data challenged the original notion that anti-RA33 autoantibodies are highly specific for RA [7]. Along with other RA-specific autoantibodies, such as rheumatoid factor (RF) and anticitrullinated peptide/protein antibodies (ACPA), these antibodies are of interest to rheumatologists as they appear to be present in early disease states, especially in RF-negative patients [8, 9]. Furthermore, they are associated with relatively mild and nonerosive disease in the absence of high-titer RF and ACPA such as anticitrullinated cyclic peptide (CCP) antibodies [8]. Recently, several anti-hnRNP autoantibodies have been investigated in patients with SARD [10]. Such a meticulous assessment concluded that the most prevalent anti-RA33 antibody by ELISA is directed against hnRNP B1.

The aim of the present study was to develop a novel ELISA detecting anti-hnRNP B1 autoantibodies and to investigate their prevalence in a Russian cohort of patients with RA and other SARD, as well as controls. As these autoantibodies are directed against a complex with pleiotropic functions, we speculated that autoreactivity against hnRNP B1 could bear pathogenic significance and it is of clinical relevance, stratifying patients according to distinct clinical phenotypes. Thus, we also attempted to correlate the occurrence of anti-hnRNP B1 autoantibodies with disease-related clinical manifestations.

## 2. Patients and Methods

### 2.1. Patients

In total, 397 patients with SARD and 174 controls were enrolled in the study. Characteristics of patients and controls are outlined in Table 1. Patients with SARD consisted of 165 patients with RA, 58 patients with spondyloarthropathy (SPA), 42 patients with juvenile chronic arthritis (JCA), 50 patients with SLE, 65 patients with systemic sclerosis (SSc), and 17 patients with Sjögren's syndrome (SS). Diagnosis of SARD had been established based on typical clinical, biochemical, histological, and serological features according to the criteria of the respective classification criteria of each SARD. Controls consisted of 52 hyperlipidemic donors in whom there was no current evidence or past medical history of SARD. Furthermore, 122 blood donors were included in the control group (Table 1).

The study was approved by the ethics committee of Almasov's Centre, St. Petersburg, vote number 12421, May 2012. Aliquots of the sera stored at −20°C were used for the study of antibody reactivity.

### 2.2. Assessment of Vascular Stiffness

Measurement of vascular stiffness by pulse wave velocity (PWV) and augmentation index (AI) was performed using applanation tonometry with the SphygmoCor system (AtCor Medical Pty Ltd., Sydney, Australia). Briefly, PWV and AI adjusted to a heart rate of 75 beats per minute were registered in subgroups of patients with late RA, SSs, SPA, and HYI as cardiology controls. Pulse wave velocity was assessed in patients after 15 minutes of rest in a sitting position. Measurements were done consequentially above carotid and femoral arteries during 10 seconds with simultaneous registration of electrocardiography, which was used to determine the moment of heart contraction. Distance of pulse wave propagation from carotid arteries to femoral arteries was measured directly and divided by the time of wave propagation to calculate pulse wave velocity. The reference ranges for PWV were defined as less than 10 meters per second.

Augmentation index was calculated from the difference between first and second wave of pulse pressure expressed as a percentage of pulse pressure. Pulse pressure was registered with pinpoint probe over ulnar artery during 10 seconds.

### 2.3. Detection of Anti-hnRNP B1 Autoantibodies by ELISA

Anti-hnRNP B1 IgG was assessed in serum samples of patients and controls by an ELISA. This assay employs recombinant human hnRNP B1 expressed in* E. coli* (in.vent DIAGNOSTICA GmbH, Hennigsdorf, Germany). Briefly, hnRNP B1 at a concentration of 5 mg/L was coated onto the solid phase of Maxisorb microtiter plates (Thermo Scientific Inc./Nunc, Germany) in bicarbonate buffer, pH 9.5, at 4°C for 26 h. After blocking with 0.05 mol/L Tris-HCl and 1% bovine serum albumin (TrisBSA, pH 7.4) at room temperature (RT) for 1 h, serum samples diluted 1 : 100 in TrisBSA were incubated at RT for 1 h and washed. Horseradish peroxidase-conjugated anti-human IgG was added and developed with ready-to-use H_2_O_2_/TMB substrate. The reaction was stopped with 0.25 mol/L sulphuric acid after 15 min. The optical density (OD) of the samples was read using a microplate reader (BioTek Instruments Inc., Winooski, USA) at a wavelength of 450 nm against 620 nm and results were expressed as arbitrary units (U/mL). The cut-off for positivity at 10 U/mL determined by receiver operating characteristics curve analysis was used. The functional assay sensitivity [11] representing the lowest antibody concentration with a coefficient of variation smaller than 20% was determined at 6.4 U/mL. The intra- and interassay variances were determined at 4% and 6%, respectively, employing a serum with a concentration of 12.0 U/mL anti-hnRNP B1 antibody. Testing 299 sera of patients suffering from RA, anti-hnRNP B1 antibody analysis by the novel ELISA was correlated with anti-hnRNP A2 (RA33) antibody detection by a commercially available ELISA (HUMAN, Wiesbaden, Germany) (see Supplementary Figure 1 in Supplementary Material available online at http://dx.doi.org/10.1155/2014/516593). There was a weak, yet significant, correlation between both anti-hnRNP antibody assays (Spearman's rho = 0.209, 95% confidential interval [CI]: 0.098–0.315; *P* < 0.001). In contrast to the commercially available assay, the novel ELISA demonstrated no significant difference in anti-hnRNP antibody levels testing fresh and unthawed aliquots of long-term stored sera.

To confirm the specificity of anti-hnRNP B1 antibody detection by the novel ELISA, 5 sera of patients suffering from rheumatoid arthritis and systemic sclerosis each demonstrating anti-hnRNP B1 IgG positivity were tested by immunoblot employing the recombinant hnRNP B1 polypeptide. All 10 sera demonstrated a clear positive reaction to hnRNP B1 blotted onto a nitrocellulose membrane (Supplementary Figure 2).

### 2.4. Detection of RA-Specific Autoantibodies

Rheumatoid factor was determined with Tina-Quant immunoturbidimetry assay (Roche Diagnostics/Roche Deutschland Holding GmbH, Penzberg, Germany) and results were expressed in IU/mL. Concentrations over 15 IU/mL were scored positive. Anti-CCP IgG was determined by ELISA according to the instructions of the manufacturer (Euroimmun AG, Lübeck, Germany). The OD was read in a microplate reader at 450 nm and results were expressed as relative units (RU/mL). The cut-off for positivity at 5 RU/mL in accordance with the recommendations of the manufacturer was used for these assays.

### 2.5. Statistical Analysis

A Kolmogorov-Smirnov test was used to analyse the data for normality. The measured values were expressed as medians with 95% CI. The two-tailed, nonparametric Mann-Whitney and Kruskal-Wallis tests were used to test for statistically significant differences of independent samples in 2 or more groups, respectively. The nonparametric Wilcoxon test was employed to test paired samples.

Spearman's rank correlation test was applied for within-group comparison. Comparison of prevalence rates between groups was performed by two-tailed Fisher's exact test. *P* values less than 0.05 were considered significant. Calculations were performed using Medcalc statistical software (Medcalc, Mariakerke, Belgium).

## 3. Results

### 3.1. Anti-hnRNP1 B1 Autoantibodies in SARD

Elevated anti-hnRNP B1 autoantibodies have been found to be significantly more prevalent in patients with SARD (47/397, 11.8%) in comparison with controls (2/174, 1.1%) (*P* < 0.001, Table 2). Amongst SARD patients, those with RA (24/165, 14.5%), SPA (6/58, 10.3%), SSc (11/65, 16.9%), and SLE (4/50, 8.0%) demonstrated significantly higher prevalences of anti-hnRNP 1 autoantibodies compared to controls (*P* < 0.001, *P* = 0.004, *P* < 0.001, and *P* = 0.023, resp.).

**Figure 1:**
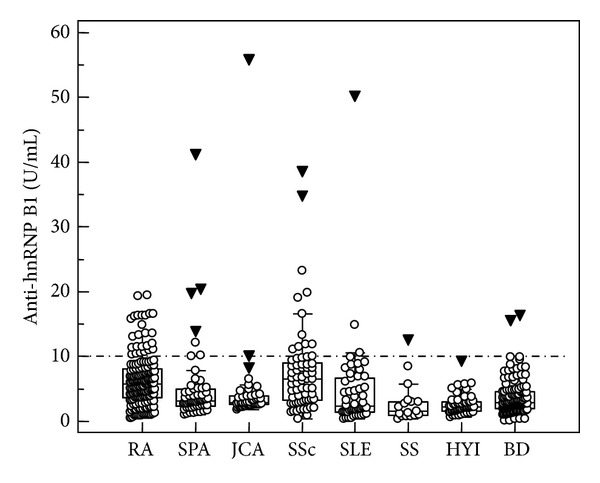
Anti-hnRNP B1 IgG levels in 165 patients (pts) with rheumatoid arthritis (RA), 58 pts with spondyloarthropathy (SPA), 42 pts with juvenile chronic arthritis (JVA), 65 pts with systemic sclerosis (SSc), 50 pts with systemic lupus erythematosus (SLE), 17 pts with Sjögren's syndrome (SS), 52 controls with hyperlipidemia, and 112 blood donors (BD) detected by enzyme-linked immunosorbent assay (ELISA). (Data are displayed as U/mL in Box-and-Whisker plots with far-out values, defined as values that are smaller than the lower quartile minus 3 times the interquartile range or larger than the upper quartile plus 3 times the interquartile range, displayed as solid triangles).

Anti-hnRNP B1 autoantibody levels differed significantly amongst the 397 patients with SARD and the 174 controls (ANOVA, Kruskal Wallis test, *P* < 0.001) (Figure 1). Amongst patients with SARD, the highest anti-hnRNP B1 autoantibody concentrations were found in patients with RA (median: 5.8, interquartile range [IQR]: 3.6–8.1) and SSc (median: 6.5, IQR: 3.2–9.0) which differed significantly from the other patient groups (SPA, JCA, SLE, and SS, *P* < 0.001, resp.) and controls (HLI and BD, *P* < 0.001, resp.). However, patients with RA and SSc did not reveal significantly different anti-hnRNP B1 autoantibody levels (*P* > 0.05).

### 3.2. Anti-hnRNP1 B1 Autoantibodies in RA

Rheumatoid arthritis-specific RF showed a prevalence of 118/165 (71.5%) in serum samples of 165 patients with RA, whereas antibodies against CCP were elevated in 126/165 (76.4%) sera thereof (Table 3). There was no correlation between anti-hnRNP autoantibody levels and those of RF and anti-CCP antibody according to rank correlation analysis (Table 4). Furthermore, neither in RF-positive compared with RF-negative patients nor in anti-CCP antibody positives against anti-CCP antibody negatives were anti-hnRNP B1 autoantibodies significantly different (*P* > 0.05, resp.). There was also no significant difference in RF and/or anti-CCP antibody positives compared with the respective negatives (*P* > 0.05).

Interestingly, a significant negative correlation of anti-hnRNP B1 autoantibody with disease duration could be established hinting at an early occurrence thereof in RA patients. However, there was no significant difference of anti-hnRNP autoantibody levels as well as prevalence in early (disease duration of less than 12 months, *n* = 102) and established RA (*P* > 0.05). Although anti-hnRNP B1 autoantibodies correlated significantly with C-reactive protein (CRP) levels, erythrocyte sedimentation rate (ESR), and joint space narrowing of hands, there was no significant association with the disease activity score of 28 joints (DAS28) in the patients with RA.

### 3.3. Anti-hnRNP1 B1 Autoantibodies in SSc

To investigate the association with clinical phenotype of patients with SSc, clinical characteristics thereof have been obtained (Table 5). In contrast to patients with RA, there was no significant correlation between anti-hnRNP B1 autoantibody and ESR, CRP levels, and duration of disease (*P* > 0.05). Furthermore, no significant correlation of autoreactivity to hnRNP B1 with fibrotic clinical manifestations in SSc such as lung and skin involvement could be established. Given the relative small number of the sera tested from patients with SSc, a safe conclusion cannot be reached and larger studies are warranted. However, like in patients with RA, there was a significantly positive correlation with the age of patients in this group. Interestingly, a significantly positive correlation with the presence of clinical manifestations including digital ulcers and esophagitis could be established (*P* = 0.007, *P* = 0.016). Notably, anti-hnRNP B1 autoantibodies demonstrated an association with hypertension and associated features such as arterial wall elasticity and pulse wave velocity (PWV) (*P* = 0.009, *P* = 0.009, and *P* = 0.004, resp.). Preliminary assessment revealed a noteworthy positive correlation of anti-hnRNP B1 autoantibodies with PWV in patients with RA (*n* = 39, Spearman's rho = 0.41; *P* = 0.009) but not in patients with SPA and HYI (*P* > 0.05, resp.).

## 4. Discussion

Loss of immune tolerance to components of large RNP moieties being part of spliceosomes or ribosomes seems to be characteristic for distinct SARD [2]. In particular, autoantibodies against the hnRNP complex composed of pre-mRNA and approximately 30 different proteins have been the interest of rheumatologists as putative serological markers in SARD [10, 12]. Autoantibodies to hnRNP A2 have been described in patients with RA and are thought to be associated with milder disease [9, 13]. These autoantibodies have been proposed for autoantibody profiling in RA serological testing, as they do not seem to correlate with RF or APCA [8, 14]. Autoantibody profiling appears to be a sensible approach in the serology of SARD supported by novel developments in the modern autoimmunity laboratory addressing the need of analyzing several autoantibodies simultaneously [8, 15–19].

A comprehensive clinical study has shown that anti-hnRNP B1 autoantibodies interacting with an alternatively spliced variant of hnRNP A2 are most prevalent in patients with SARD amongst 10 different anti-hnRNP autoantibodies detected by ELISA [10]. Nevertheless, autoreactivity to hnRNP A2 does not appear to be different to that against its alternatively spliced variant B1 [7]. Thus, the present clinical study investigated anti-hnRNP B1 autoantibody levels in 397 Russian patients with SARD and 174 controls. Significantly higher anti-hnRNP B1 autoantibody prevalences and levels were found in patients with RA and SSc. Interestingly, an overlap syndrome of RA and limited SSc has been described, which was characterized by an incomplete CREST syndrome and cross-reactivity of anticentromere with anti-hnRNP B1 autoantibodies [20, 21]. Recently, an association of anti-hnRNP autoantibodies with erosive arthritis has been described in patients with SSc [22]. Thus, anti-hnRNP autoantibody might become a nonspecific but useful marker for joint involvement in SSc patients and identify SSc patients prone to develop joint damage. In general, the radiological articular manifestations in SSc are less severe compared to those noted in patients with RA [23]. We did not find a significant correlation of articular manifestations with anti-hnRNP B1 autoantibody levels in patients with SSc.

However, in contrast to Op de Beéck et al., Russian patients with SARD demonstrated a lower prevalence of anti-hnRNP autoantibodies in patients with SARD in general [10]. In particular, Russian patients with SS and SLE showed a comparatively low prevalence (5.9%* versus* 44.1% and 8.0%* versus* 37.1% in [10], resp.). These differences could be due to the different patient characteristics of the respective cohorts or different assay performance of the ELISA employed for autoantibody testing. Indeed, detection of autoantibodies to hnRNP seems to require the preservation of conformational epitopes which can be influenced by the recombinant expression system and coating procedure for ELISA-solid phases utilized as demonstrated for other autoantigenic targets as well [24, 25]. Furthermore, Russian patients with RA can demonstrate different prevalences of RA-specific autoantibodies than reported in studies with Caucasian patients [26].

As a matter of fact, hnRNPs have important cellular functions and their respective autoantibodies could alter their functional properties [1, 3, 27]. This has led us to speculate that, in addition to their diagnostic relevance, anti-hnRNP autoantibodies may bear pathogenic potential. Thus, we attempted to correlate the loss of tolerance to hnRNP B1 with clinical characteristics in Russian patients with RA and SSc. In this study, anti-hnRNP B1 autoantibodies were not associated with disease activity or erosions in patients with RA. These findings support the assumption that anti-hnRNP antibodies are more frequent in RA patients with mild disease compared to those with more active disease [8, 9]. Interestingly, there was a positive correlation with clinical manifestations of SSc such as the occurrence of esophagitis and digital ulcers. Furthermore, an association with hypertension and arterial wall elasticity, as well as PWV, that is, features of arterial stiffness, could be established. Arterial wall stiffness is recommended as a risk factor for cardiovascular events in patients with arterial hypertension by the European Network for Non-Invasive Investigation of Large Arteries [28]. It needs to be noted that there is accumulating evidence of increased arterial stiffness in patients with SSc [29, 30]. Our preliminary results revealed a positive correlation of anti-hnRNP B1 autoantibody with PWV in RA patients, but this finding requires external validation in larger cohorts. Nevertheless, such a correlation was not seen in hyperlipidemic patients with an increased risk for atherosclerosis and arterial stiffness. To the best of our knowledge, this is the first report of a significant association of anti-hnRNP B1 autoantibodies with hypertension in SARD and could support the existence of an overlap between RA and SSc [20].

In summary, anti-hnRNP B1 autoantibodies occur in Russian patients with SARD and particularly in patients with RA and SSc. In the latter patient groups, they seem to be correlated with clinical characteristics such as hypertension. Larger prospective studies are urgently warranted to address the clinical relevance and the pathogenic significance of these autoantibodies.

## Supplementary Material

Comparison of the novel anti-hnRNP B1 enzyme-linked immunosorbent assay (ELISA) with a commercially available ELISA detecting anti-hnRNP A2 (RA33) antibodies and an immunoblot assay analyzing anti-hnRNP B1 antibodies
For the correlation of anti-hnRNP B1 antibody analysis by the novel ELISA with anti-hnRNP A2 (RA33) antibody detection by a commercially available ELISA (HUMAN, Wiesbaden, Germany), 299 sera of patients suffering from rheumatoid arthritis (RA) were tested by both methods (Supplementary Figure 1). There was a weak, yet significant, correlation between both anti-hnRNP antibody assays (Spearman's rho = 0.209, 95% confidential interval: 0.098 – 0.315; *P*< 0.001).
To confirm the specificity of anti-hnRNP B1 antibody detection by the novel ELISA, 10 sera of 5 patients suffering from RA and 5 patients with systemic sclerosis (SSc) each demonstrating anti-hnRNP B1 IgG positivity were tested by immunoblot assay employing the recombinant hnRNP B1 polypeptide. All 10 sera demonstrated a clear positive reaction of IgG to the hnRNP B1 polypeptide blotted onto a nitrocellulose membrane (Supplementary Figure 2).

## Figures and Tables

**Table 1:** Characteristics of individuals studied including 397 patients with systemic autoimmune rheumatic diseases and 174 controls.

Diagnosis	*n*	Gender f/m	Age median	Age IQR	DD median	DD IQR	Clinical characteristics
RA	165	102/63	54.0	47.0–60.0	0.58	0.3–5.0	Diagnosis was based on ACR/EULAR 2010 criteria, median DAS28: 4.3 (IQR 3.3–5.2)

SPA	58	17/41	37.0	32.0–50.0	7.0	4.5–12.0	52 pts with ankylosing spondylitis according to the New York criteria and 6 pts with axial spondyloarthritis according to the ASAS criteria 2010

JCA	42	26/16	10.6	6.9–15.4	4.4	1.0–9.9	12 pts with polyarticular disease, 24 pts with oligoarticular disease, 6 pts with systemic disease

SLE	50	47/3	36.0	27.0–45.0	5.0	3.0–12.0	Diagnosis was based on ACR 1997 Revisited criteria, median SLEDAI 4.0 (IQR 2.0–8.0)

SSc	65	62/3	53.0	42.0–60.0	5.0	3.0–9.0	Diagnosis was based on ACR (ARA) criteria 1980 Rodnan skin involvement score: median 16 (IQR 9–22), 31 pts with diffuse scleroderma, 27 pts with limited scleroderma, 7 pts with overlap syndrome

SS	17	17/0	62.5	55.0–66.0	5.0	4.0–11.0	Diagnosis was based on American-European classification criteria, median ESSPRI: 5.3 (IQR 3.8–7.0)

HYI (cardiology control)	52	33/19	52.0	44.0–55.0			Clinically observed for absence of rheumatic diseases

BD	122	83/39	33.0	29.5–42.0			

ACR: American College of Rheumatology; DAS28: disease activity score of 28 joints; DD: disease duration; ESSPRI: The EULAR Sjögren's Syndrome Patient Reported Index; EULAR: European League Against Rheumatism; HYI: hyperlipidemic individuals with high Framingham cardiovascular score; JCR: juvenile chronic arthritis; *n*: number; pts: patients; RA: rheumatoid arthritis; SLE: systemic lupus erythematosus; SLEDAI: systemic lupus erythematous disease activity index; SPA: spondyloarthropathy; SS: Sjögren's syndrome; SSc: systemic sclerosis.

**Table 2:** Prevalence of anti-hnRNP B1 autoantibodies in sera of 397 patients with systemic autoimmune rheumatic diseases and 174 controls.

	RA *n* = 165	SPA *n* = 58	JCA *n* = 42	SSc *n* = 65	SLE *n* = 50	SS *n* = 17	SARD *n* = 397	HYI *n* = 52	BD *n* = 122	Controls *n* = 174
Positives, >10 U/mL	24 (14.5%)	6 (10.3%)	1 (2.4%)	11 (16.9%)	4 (8.0%)	1 (5.9%)	47 (11.8%)	0 (0.0%)	2 (1.6%)	2 (1.1%)

HYI: hyperlipidemic individuals with high Framingham cardiovascular score; JCR: juvenile chronic arthritis; *n*: number; RA: rheumatoid arthritis; SLE: systemic lupus erythematosus; SPA: spondyloarthropathy; SS: Sjögren's syndrome; SSc: systemic sclerosis.

**Table 3:** Anti-hnRNP B1 autoantibodies in 165 patients with rheumatoid arthritis depending on the presence of rheumatoid factor and anti-CCP antibodies.

	RF		Anti-CCP		RF/anti-CCP	
	Positive (*n* = 118)	Negative (*n* = 47)	Positive (*n* = 126)	Negative (*n* = 39)	Positive (*n* = 102)	Negative (*n* = 23)

*n* >10 U/mL	17 (14.4%)	7 (14.9%)	17 (13.5%)	7 (17.9%)	14 (13.7%)	4 (17.4%)

CCP: citrullinated cyclic peptide; RF: rheumatoid factor.

**Table 4:** Correlation of anti-hnRNP B1 autoantibodies with clinical characteristics of patients with RA.

	Valid number of patients	Spearman	*t* (*N* − 2)	*P*
Age	161	0.229447	2.97253	0.003
Duration of disease	144	−0.259660	−3.20410	0.002
ESR	156	0.197243	2.49678	0.014
CRP	157	0.259968	3.35182	0.001
DAS28	120	−0.128757	−1.41040	0.161
Smoking habit	108	−0.120022	−1.24470	0.216
Anti-CCP antibody	108	0.105416	1.09141	0.278
RF	157	0.027336	0.34046	0.734
Joint space narrowing hand	75	0.309004	2.77598	0.007
Joint space narrowing feet	74	0.212152	1.84210	0.070
Erosion feet	74	0.156521	1.34470	0.183

CRP: C-reactive protein; ESR: erythrocyte sedimentation rate; CCP: citrullinated cyclic peptide; DAS28: disease activity score of 28 joints; RF: rheumatoid factor.

**Table 5:** Correlation of anti-hnRNP B1 autoantibodies with clinical characteristics of patients with SSc.

	Valid number of patients	Spearman	*t* (*N* − 2)	*P*
Age	64	0.314713	2.61071	0.012
Duration of disease	64	−0.022761	−0.179267	0.858
Hypertension presence	60	0.333848	2.69726	0.009
Duration of hypertension	54	0.352815	2.71904	0.009
Augmentation index (arterial wall elasticity)	54	0.350336	2.69725	0.009
PWV	53	0.390139	3.02593	0.004
Digital ulcers	60	−0.347099	−2.81867	0.007
Esophagitis	59	−0.312778	−2.48616	0.016
Right ventricle dimensions	58	0.271581	2.11169	0.039
Heart block	57	0.254881	1.95481	0.056
ESR	62	0.236032	1.88146	0.065
CRP	59	0.218667	1.69184	0.096

CRP: C-reactive protein; ESR: erythrocyte sedimentation rate; CCP: citrullinated cyclic peptide; PWV: pulse wave velocity.

## Abbreviations

ACPA: Anticitrullinated peptide/protein antibody
AI: Augmentation index
BD: Blood donors
CCP: Citrullinated cyclic peptide
CRP: C-reactive protein
DAS28: Disease activity score of 28 joints
ELISA: Enzyme-linked immunosorbent assay
ESR: Erythrocyte sedimentation rate
HYI: Hyperlipidemic individuals
hnRNP: Heterogeneous nuclear ribonucleoprotein
IU: International units
JCR: Juvenile chronic arthritis
PWV: Pulse wave velocity
RA: Rheumatoid arthritis
RF: Rheumatoid factor
RT: Room temperature
RU: Relative units
SARD: Systemic autoimmune rheumatic disease
SLE: Systemic lupus erythematosus
SPA: Spondyloarthropathy
SS: Sjögren's syndrome
SSc: Systemic sclerosis.
