# Supplementary material for: Anti-hnRNP B1 (RA33) Autoantibodies Are Associated with the Clinical Phenotype in Russian Patients with Rheumatoid Arthritis and Systemic Sclerosis
Source: J Immunol Res. 2014 May 4;2014:516593. doi: 10.1155/2014/516593 (PMC4027001; doi:10.1155/2014/516593)
Supplement: Supplementary file 1 — Comparison of the novel anti-hnRNP B1 enzyme-linked immunosorbent assay (ELISA) with a commercially available ELISA detecting anti-hnRNP A2 (RA33) antibodies and an immunoblot assay analyzing anti-hnRNP B1 antibodies For the correlation of anti-hnRNP B1 antibody analysis by the novel ELISA with anti-hnRNP A2 (RA33) antibody detection by a commercially available ELISA (HUMAN, Wiesbaden, Germany), 299 sera of patients suffering from rheumatoid arthritis (RA) were tested by both methods (Supplementary Figure 1). There was a weak, yet significant, correlation between both anti-hnRNP antibody assays (Spearman's rho = 0.209, 95% confidential interval: 0.098 – 0.315; P< 0.001). To confirm the specificity of anti-hnRNP B1 antibody detection by the novel ELISA, 10 sera of 5 patients suffering from RA and 5 patients with systemic sclerosis (SSc) each demonstrating anti-hnRNP B1 IgG positivity were tested by immunoblot assay employing the recombinant hnRNP B1 polypeptide. All 10 sera demonstrated a clear positive reaction of IgG to the hnRNP B1 polypeptide blotted onto a nitrocellulose membrane (Supplementary Figure 2). [file 516593.f1.pptx]

## Slide 1
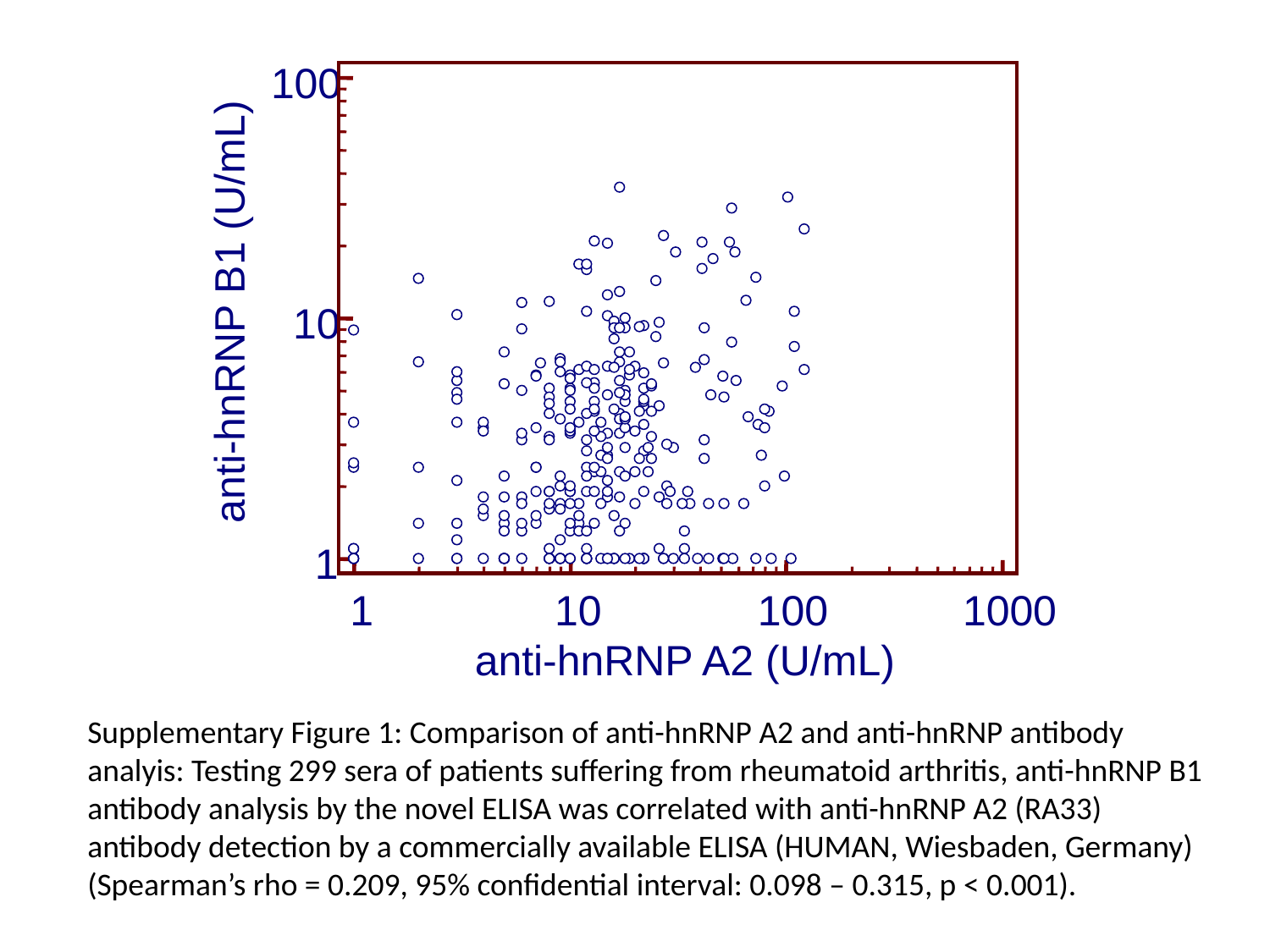

100
anti-hnRNP B1 (U/mL)
10
1
1
10
100
1000
anti-hnRNP A2 (U/mL)
Supplementary Figure 1: Comparison of anti-hnRNP A2 and anti-hnRNP antibody analyis: Testing 299 sera of patients suffering from rheumatoid arthritis, anti-hnRNP B1 antibody analysis by the novel ELISA was correlated with anti-hnRNP A2 (RA33) antibody detection by a commercially available ELISA (HUMAN, Wiesbaden, Germany) (Spearman’s rho = 0.209, 95% confidential interval: 0.098 – 0.315, p < 0.001).

## Slide 2
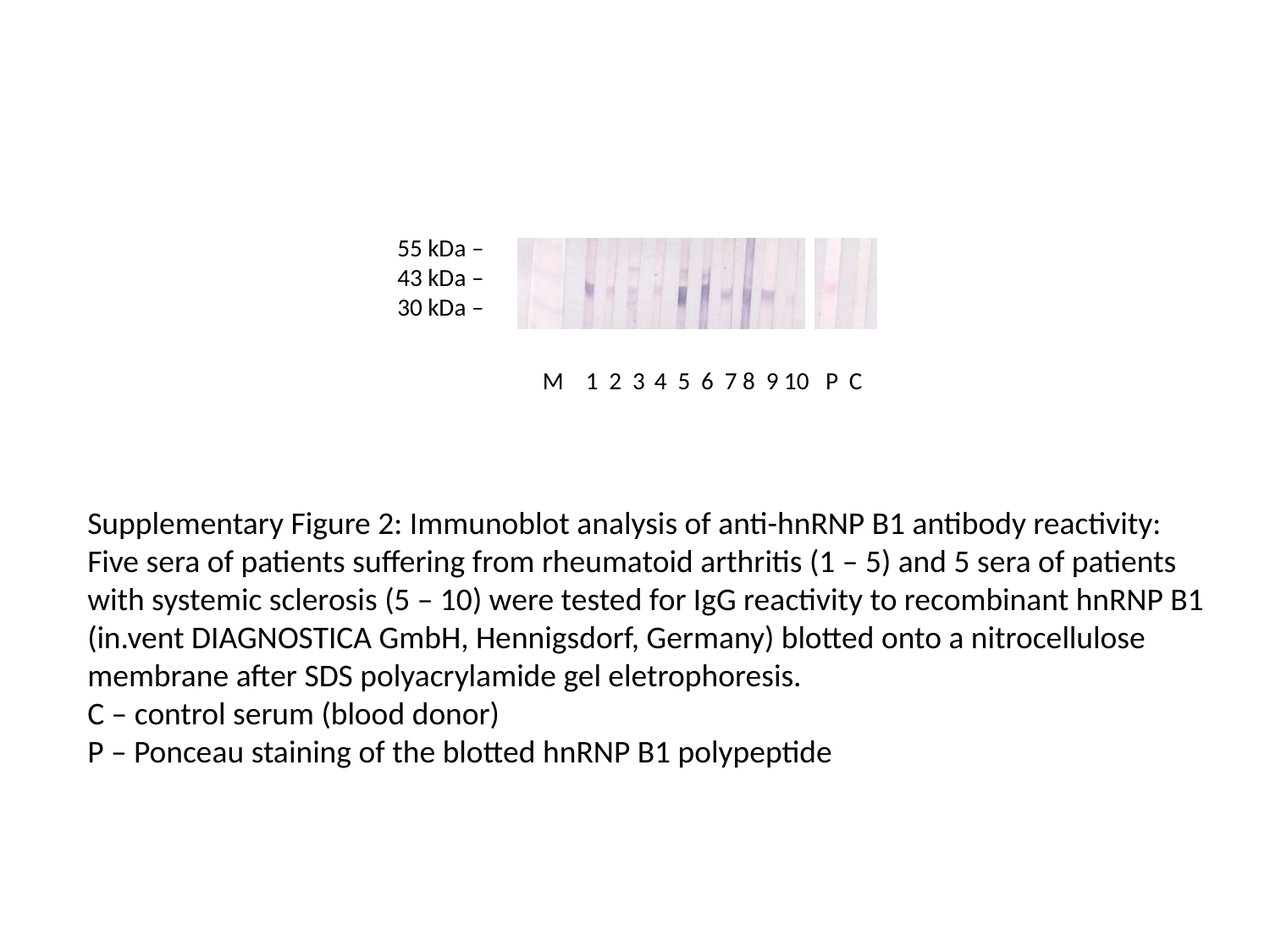

55 kDa –
43 kDa –
30 kDa –
M 1 2 3 4 5 6 7 8 9 10 P C
Supplementary Figure 2: Immunoblot analysis of anti-hnRNP B1 antibody reactivity: Five sera of patients suffering from rheumatoid arthritis (1 – 5) and 5 sera of patients with systemic sclerosis (5 – 10) were tested for IgG reactivity to recombinant hnRNP B1 (in.vent DIAGNOSTICA GmbH, Hennigsdorf, Germany) blotted onto a nitrocellulose membrane after SDS polyacrylamide gel eletrophoresis.
C – control serum (blood donor)
P – Ponceau staining of the blotted hnRNP B1 polypeptide
